# Multidisciplinary Management of the Rare Coexistence of Multiple Endocrine Neoplasia Type 1 and Chronic Thromboembolic Pulmonary Hypertension: A Case Report

**DOI:** 10.7759/cureus.114115

**Published:** 2026-08-07

**Authors:** Gita Deb, Julian Emmanuel, Jenny L Bacon, Sam Wong, Amna Hayat

**Affiliations:** 1 Medicine, East Surrey Hospital, Redhill, GBR; 2 Endocrinology and Diabetes, East Surrey Hospital, Redhill, GBR; 3 Respiratory Medicine, East Surrey Hospital, Redhill, GBR; 4 Clinical Oncology, Royal Marsden Hospital, London, GBR; 5 Radiology, East Surrey Hospital, Redhill, GBR

**Keywords:** diagnosis & prognosis, endocrine neoplasms, health-related quality of life, men1, thromboembolic disease

## Abstract

Multiple endocrine neoplasia type 1 (MEN1) is a complex endocrine tumour syndrome. Broader understanding of its diverse clinical manifestations has transformed its surveillance and management. The protracted nature of MEN1 significantly impacts quality of life, requiring careful observation, symptom control and tailored treatment decisions with coordinated, multidisciplinary team (MDT) care. This case report follows the clinical journey of a patient with MEN1 and illustrates how management can be challenging when faced with overlapping chronic conditions. In this instance, the patient’s course was further complicated by chronic thromboembolic pulmonary hypertension (CTEPH), posing additional diagnostic and therapeutic considerations. The case underscores the importance of vigilant follow-up of patients, not only due to high disease penetrance and risk of recurrence, but to avoid diagnostic overshadowing of other significant co-morbidities. With a focus on long-term outcomes and balancing invasive interventions, this case report truly demonstrates the strengths of the MDT approach in healthcare.

## Introduction

Multiple endocrine neoplasia type 1 (MEN1) is a rare genetic condition inherited in an autosomal dominant fashion, characterised by tumours most commonly affecting the pituitary gland, parathyroid glands and pancreas. Whilst deemed an infrequent disease, only affecting around 3-20 in 100,000, it is a condition not only recognised for having diagnostic complexities but also a considerable impact on life expectancy and quality of life [1,2]. The predicted life span of an individual with MEN1 is at least 10 years less than that of the general population, although data have suggested it has increased from the average of 55 years old to around 70 years old due to medical advancements [2].

Quality of life is significantly negatively impacted with MEN1. High disease penetrance (95% by the age of 40) [1] with the psychological impact of the certainty of developing particular tumours, young ages of presentation causing a significant burden of disease impact during an individual’s lifespan, and repeated hospital admissions (often with multiple surgeries) all play a part in the challenges of living with MEN1 [3].

With its various manifestations over a prolonged period of time, this case demonstrates how MEN1 can significantly impact an affected individual during their lifespan. This case report importantly highlights how vital the role of regular multidisciplinary input and follow-up is in MEN1 management to improve outcomes.

Of note, to our knowledge, it is also the first reported case describing the coexistence of MEN1 with chronic thromboembolic pulmonary hypertension (CTEPH) - another uncommon disorder which has an estimated prevalence of 19-50 per million that is characterised by the presence of pulmonary hypertension (PH) and subsequent right heart failure as a result of intravascular occlusion by fibrotic material in the pulmonary arteries [4]. Therefore, we describe the challenges of multidisciplinary management in a patient with two rare and complex conditions.

## Case presentation

The patient, now in his 70s, initially presented two decades ago with symptoms of diarrhoea and reflux. He was found to have a high calcium level of 3.97mmol/L (normal serum calcium levels are <2.6mmol/L, with severe hypercalcaemia being diagnosed if >3.5mmol/L [5]). Further investigations identified parathyroid hyperplasia with elevated parathyroid levels measuring 41.9pmol/L (normal reference range 1.1-6.9 pmol/L [6]); Zollinger-Ellison syndrome with elevated gastrin levels and evidence of severe peptic ulceration and duodenitis on endoscopy; and carcinoid syndrome with elevated chromogranin A levels whereby abdominal imaging found four lesions of the pancreas, mainly in the head and neck of the pancreas, with subsequent fine needle aspiration confirming endocrine neoplasms. Furthermore, whilst clinically and biochemically euthyroid, a technetium-99m sestamibi (99mTc-sestamibi) scan completed as part of endocrine diagnostic workup flagged a right lower lobe thyroid lesion. All of these results raised the suspicion of MEN1 syndrome. Therefore, that year, he underwent a total parathyroidectomy to treat his enlarged, overactive parathyroid gland and a right hemithyroidectomy to remove the thyroid lesion. In 2008, he then completed further surgical intervention by way of a distal pancreatectomy with splenectomy to remove his pancreatic neuroendocrine tumours. Indeed, the overarching diagnosis of MEN1 was confirmed with genetic testing in 2014.

Since then, he has remained under close surveillance by Gastroenterology and Endocrinology teams, with the management of complex diabetes (thought to be related to the pancreatic tail removal), monitoring of gut hormone profile, titration of thyroid and calcium replacement, and observation for other MEN1-related complications.

**Figure 1:**
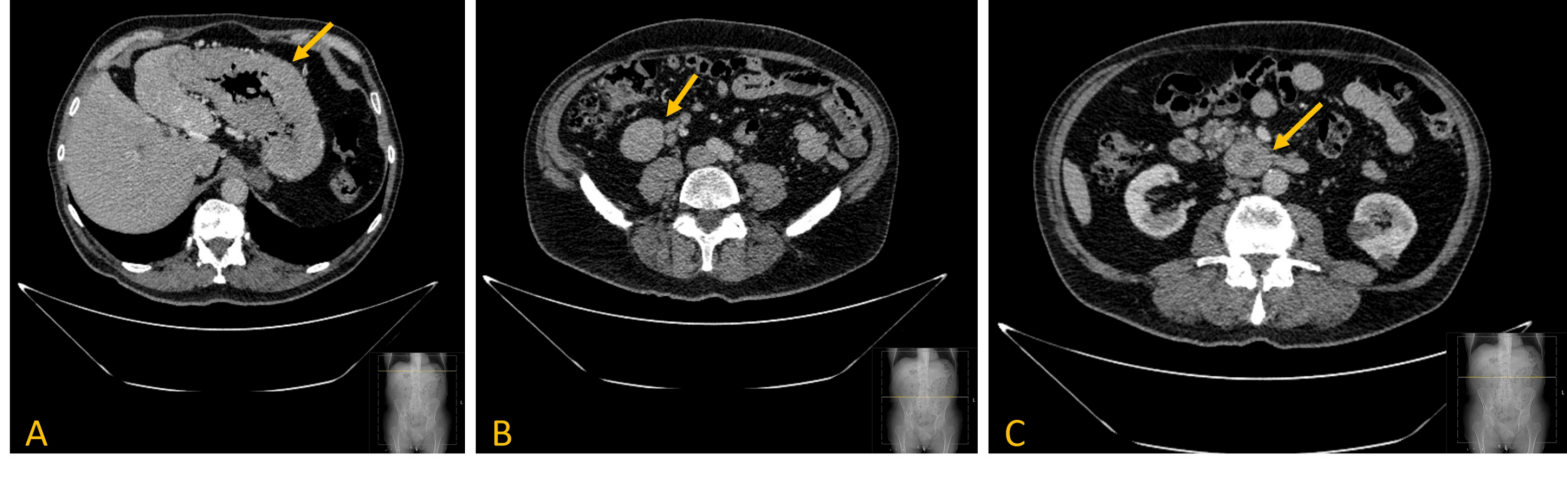
Cross-sectional CT images of the abdomen and pelvis obtained in October 2021 Image A: Cross-sectional CT image of the abdomen and pelvis demonstrating gastric wall thickening (yellow arrow). Image B: Cross-sectional CT image of the abdomen and pelvis demonstrating multiple pathological lymph nodes (yellow arrow). Image C: Cross-sectional CT image of the abdomen and pelvis demonstrating a possible duodenal mass (yellow arrow).

The patient remained stable until five years ago, when a rise in gut hormones was detected during outpatient surveillance, and a subsequent CT showed evidence of gastric wall thickening and other abnormalities (Figure 1).

This prompted endoscopic evaluation whereby an oesophagogastroduodenoscopy (OGD) detected gastric polyps; histological analysis showed the presence of a Grade 1 multifocal well-differentiated neuroendocrine tumour. As he remained asymptomatic from this point of view, he continued strict surveillance. The role of the multidisciplinary team (MDT) became crucial with the collaboration of services from Endocrinology, Gastroenterology and the specialist Neuroendocrine Tumour Team. A repeat OGD performed for progressive iron deficiency in 2025 showed oesophagitis with ulceration, moderate duodenitis with erosions and multiple polyps in the stomach, of which biopsies were taken.

**Figure 2:**
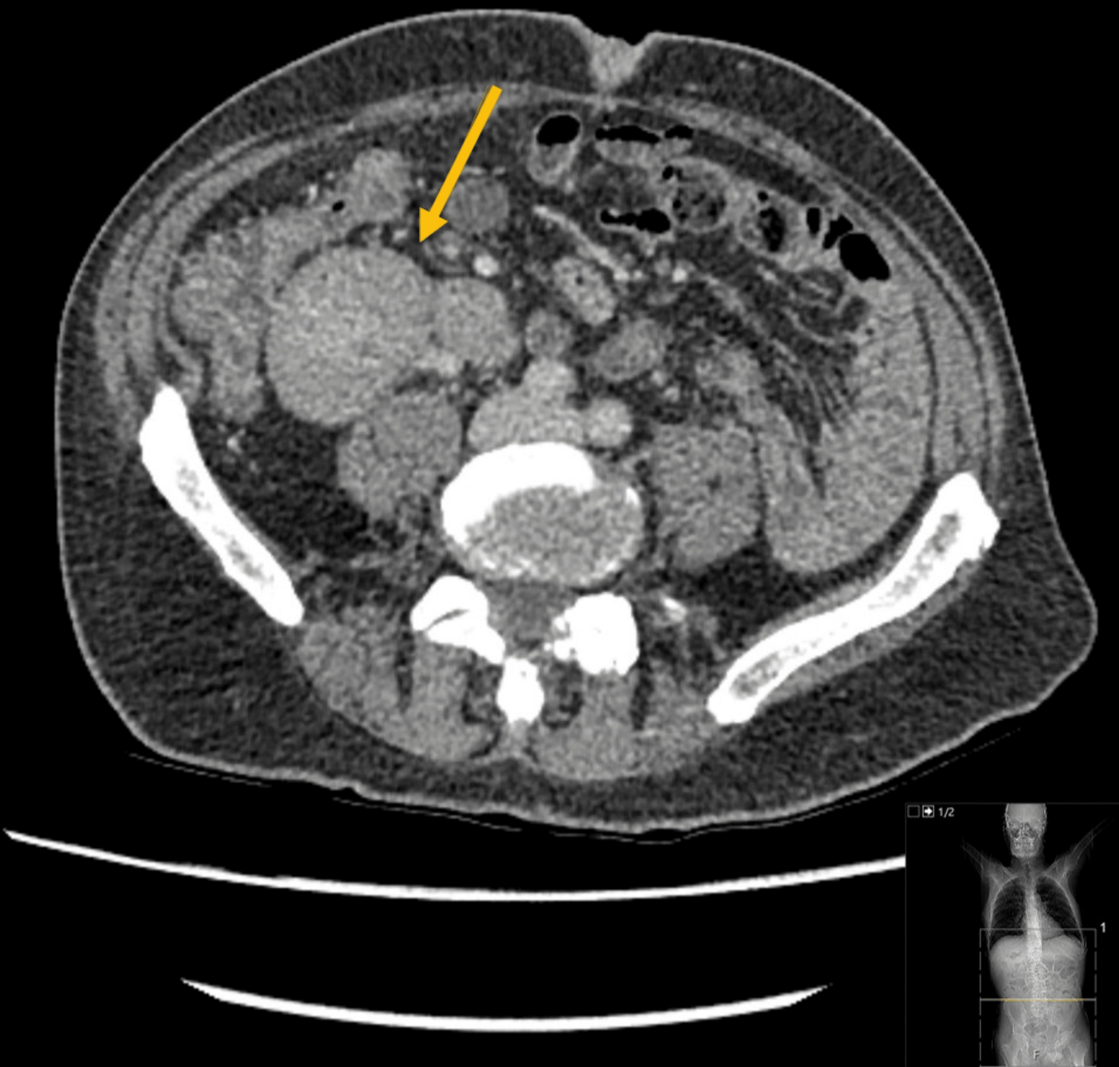
Cross-sectional CT image of the abdomen and pelvis obtained in April 2025 Cross-sectional CT image of the abdomen and pelvis demonstrating abnormally large mesenteric lymph nodes (yellow arrow).

Unfortunately, it was only when he presented to hospital in April 2025 with severe hip pain, for which he was investigated with a CT, that he was found to have abnormally large enhancing mesenteric lymph nodes in the right iliac fossa as well as multiple abnormal lymph nodes in the paraaortic and retroperitoneal regions (Figure 2).

**Figure 3:**
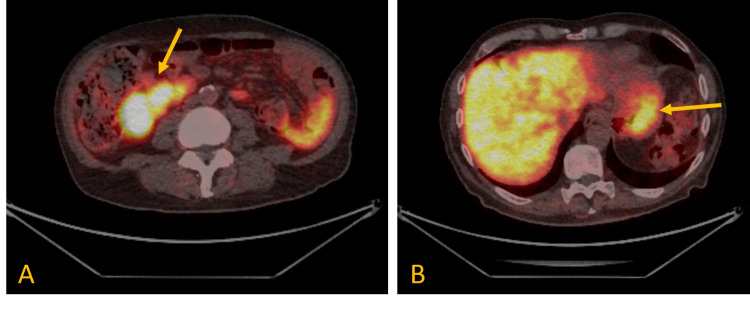
Gallium-68 DOTATATE whole-body positron emission tomography/computed tomography (⁶⁸Ga-DOTATATE PET/CT) images obtained in October 2025 Image A: Whole-body ^68^Ga-DOTATATE PET/CT image demonstrating upper abdominal adenopathy with intense DOTATATE expression (yellow arrow). Image B: Whole-body ^68^Ga-DOTATATE PET/CT image demonstrating avid uptake in the proximal stomach (yellow arrow).

This raised the concern of metastatic disease. After MDT discussion, an urgent PET scan and Oncology review were arranged. By the time of his Oncology input, his repeat biopsies had confirmed a Grade 1 well-differentiated neuroendocrine tumour in the stomach and duodenum with adenopathy in the upper abdomen. The gallium-68 DOTATATE positron emission tomography (^68^Ga-DOTATATE PET) scan demonstrated intense uptake in the abdominal lymph nodes confirming metastatic involvement (Figure 3).

Upon review of his symptoms, although he had a good performance status, consideration of his comorbidities meant that the Oncology MDT decided that focusing on tumour and symptom control with regular lanreotide injections would be the most appropriate management strategy.

In terms of his comorbidities, apart from the manifestations of MEN1 discussed thus far, the patient’s journey had recently been complicated by respiratory issues. He had initially presented to his general practitioner (GP) five years ago with chest and arm pain on a background of progressive dyspnoea for one year. Reassuringly, investigations confirmed that there were no cardiac features of his symptoms, but a later chest radiograph (CXR) showed abnormalities that were further investigated with CT scans and PET imaging. These studies noted nodules that were found to grow and disappear over the course of their surveillance, and on one occasion, when a biopsy was arranged, the lesion had resolved by the day of the procedure, so the biopsy was cancelled.

Unfortunately, the patient’s breathlessness continued to plague him; he went on to have lung spirometry, which was normal, but it did show an abnormal reduced transfer factor of the lung for carbon monoxide (% predicted TLCO). An echocardiogram showed good left ventricular function but suggested the presence of PH. A ventilation/perfusion scan was subsequently arranged, and interestingly, this flagged up perfusion defects in keeping with chronic pulmonary emboli. In light of these findings, a direct oral anticoagulant (DOAC) was started in early 2024. Despite anticoagulation, his pulmonary emboli persisted. A right heart catheter was arranged, which demonstrated persistent PH (mean pulmonary pressure 36mmHg, mean pulmonary wedge pressure 9mmHg, pulmonary vascular resistance 6 Wood Units), and therefore, a diagnosis of CTEPH was made.

He remained under the care of the Complex Pulmonary Vascular Clinic where communication with a specialist tertiary centre was initiated with regard to management options. Due to distal disease and out-of-proportion symptoms to CTEPH severity, a trial of medical management was decided by the tertiary MDT after multiple discussions balancing risks and benefits of initiating invasive management in a patient whose anaesthetic and operative risks were deemed high due to his coexisting MEN1 condition and complications. Therefore, he commenced riociguat medication in December 2025 over undergoing major surgery in the form of a pulmonary thromboendarterectomy (PEA) or less invasive surgery comprising a balloon pulmonary angioplasty (BPA). Interestingly, his symptoms were already improving after anaemia stabilisation and lanreotide treatment. There were a small number of targets identified by the MDT for potential BPA, but after further collaboration, the forward plan is to consider if BPA is necessary as a second-line approach, depending on his treatment responses to riociguat and the management of his metastatic neuroendocrine tumour with monthly lanreotide injections. Understandably, there is a prime focus on ensuring there is stability and medical optimisation before initiating any invasive care.

**Figure 4:**
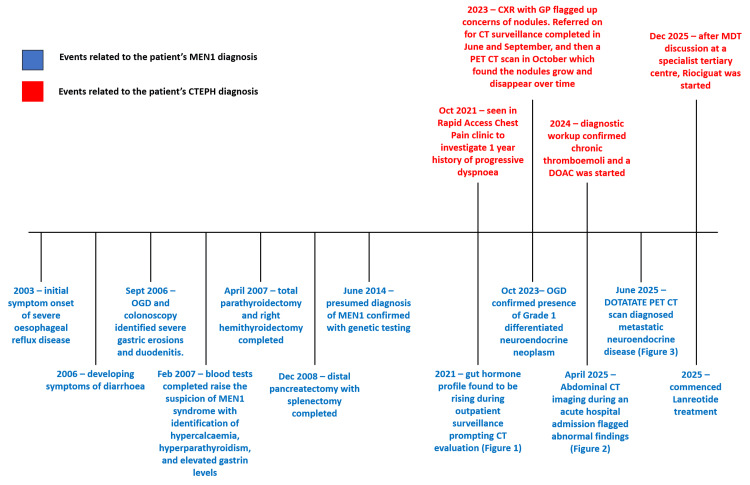
Timeline of key events in the patient’s journey A chronological timeline summarising key investigations, diagnoses and treatments during the patient’s journey spanning over 20 years, whereby events in blue depict the journey of the patient’s MEN1 diagnosis and the events in red depict the journey of the patient’s CTEPH diagnosis. MEN1: multiple endocrine neoplasia type 1; CTEPH: chronic thromboembolic pulmonary hypertension; CXR: chest X-ray; GP: general practitioner; PET: positron emission tomography; CT: computed tomography; MDT: multidisciplinary team; DOAC: direct oral anticoagulant; OGD: oesophagogastroduodenoscopy

Whilst the specialities are working closely in parallel to maintain the patient’s quality of life and symptom control, with his current MEN1 and CTEPH disease burden, it remains a complex multidisciplinary journey (Figure 4) in an elderly patient.

Outcome and follow-up

Below, the treatment paths for the patient categorised into systems are summarised:

Respiratory

There was a delay in diagnosing pulmonary emboli, which was further investigated once signs of PH and a reduced gas transfer were highlighted. Once diagnosed with pulmonary emboli, and then eventually CTEPH, there was initiation of anticoagulation in the form of a DOAC. His CTEPH was managed with riociguat, and he is awaiting follow-up to review his response to medical therapy.

Gastroenterology/Neuroendocrine

When initially diagnosed with Zollinger-Ellison and carcinoid syndrome, a high-dose proton pump inhibitor was used for symptom control until eventual surgical management with a distal pancreatectomy and splenectomy. When later diagnosed with metastatic Grade 1 multifocal well-differentiated neuroendocrine tumour, he started monthly lanreotide injections.

Endocrine

Naturally, with MEN1 being a condition that predominantly affects the endocrine organs, the need for close hormone and electrolyte monitoring is predictable.

Post-total parathyroidectomy for his initial presentation of MEN1, regular titration of thyroid, calcium and vitamin D replacement was established. Over the years, the patient has been alternating between hypercalcaemia and hypocalcaemia, which has been found to be dependent on vitamin D supplementation, calcitriol doses and sun exposure. Without usual internal regulatory mechanisms due to resection of his parathyroid glands, titration of replacement has been vital in maintaining calcium homeostasis.

There is ongoing management of his complex diabetes (type 2 diabetes mellitus, but with a history of previous pancreatic tail removal, there may be overlap with type 3c diabetes); however, insulin initiation has been avoided as he manages well with metformin and linagliptin for glycemic control.

Haematology

Regular intravenous iron or blood transfusions are provided as indicated for symptomatic iron deficiency anaemia.

## Discussion

Mutations in the MEN1 tumour suppressor gene, found on chromosome 11q13, cause a syndrome characterised by hyperplasia or tumourous growth of tissues. Historically, clinical identification by the primary organs affected led to the "3 P’s" categorisation of this disorder: the combined occurrence of parathyroid gland hyperplasia, anterior pituitary tumours and duodeno-pancreatic neuroendocrine tumours [7]. However, since the syndrome’s first description in 1903, and its phenotypic and genetic definition following on in 1953, with the eventual breakthrough of the MEN1 gene discovery in 1997, knowledge surrounding this rare condition has broadened [3]. There is better understanding of MEN1’s other clinical manifestations, such as tumours affecting over 20 other endocrine organs or even non-endocrine tissues [1], and of its molecular and genetic pathogenesis whereby now over 1500 mutations affecting the MEN1 gene and its subsequent MENIN protein product have been identified [3] - this has contributed to ever-evolving concepts of the use of genetic analysis in diagnostic and therapeutic pathways.

Importantly, in those with germline inactivating mutations of MEN1, 90% of cases are inherited, and so 10% of cases occur due to de novo mutations [3,7,8]. Therefore, in the context of understanding the implications of the diagnosis, this highlights the importance of having a comprehensive understanding of the clinical syndrome to facilitate early recognition of the condition.

In this case report, the patient had formal genetic testing, which indeed confirmed the presence of a missense mutation in exon 2 of the MEN1 gene (pP12L). Whilst he did not have any family history suggestive of familial MEN1 syndrome, identification of the mutation has allowed his family to access the necessary screening services, which is key as first-degree relatives harbour a 50% risk of also being affected. However, it is worth noting that there is no consistent correlation between genotype and phenotype; therefore, understanding the specific nature of the genetic mutation present, such as its type or location, does not predict the clinical manifestations or disease burden that may result, nor does it aid clinicians in risk stratification. Nevertheless, it is well recognised that genetic analysis has opened the door to earlier discovery of affected patients and a subsequent reduction in morbidity and mortality as reflected in the improved life expectancy of MEN1 patients since its first description [7,9].

Once correctly diagnosed, strict periodic surveillance of high-risk or affected MEN1 individuals is recommended to allow early tumour detection. Genetic analysis should be offered to all first-degree relatives, regardless of symptomatology. Then, a comprehensive MDT approach to long-term screening, surveillance and treatment is key in realistically achieving the overarching goal of management, which is of maintaining an adequate quality of life [9,10].

Importantly, clinical guidelines have recently been expanded and optimised as of 2025, with two notable groups collating further important recommendations for clinicians [11,12]. Experts in the field of MEN1, spanning multiple specialities, have reviewed key evidence and literature available on this rare diagnosis to condense guidelines into a more complete, practical and standardised strategy for relevant clinicians [11].

With regard to the patient discussed in this case, he most definitely did not lack in MDT input and indeed accessed the expertise of a specialist neuroendocrine team. This is in line with the general recommendations for MEN1 as per the available guidelines and positively impacted the patient’s overall journey due to the ability to access the relevant screening tools and speciality advice to inform his invasive treatment. Once the co-existent diagnosis of CTEPH became clear, which likely had prompt management input through his access to key specialist clinicians and secondary care pathways, the MDT was well established in communicating a treatment plan that would consider all of his co-morbidities.

Malignancy is a known risk factor for CTEPH [13], and this case report demonstrates some key diagnostic tools available to clinicians when clarifying the aetiology of PH. Therefore, considering the malignant processes in MEN1, whilst no case report has described the coexistence of MEN1 and CTEPH, it is compatible that MEN1 was an important contributory risk factor for the development of his PH.

Importantly, the investigative and therapeutic algorithms available for PH and its classified subsets are well established in the 2022 European Society of Cardiology (ESC)/European Respiratory Society (ERS) Guidelines. CTEPH is a disease that has three main treatment options performed at specialist centres, whilst always continuing appropriate anticoagulation to prevent further embolisms: medical management with advanced pulmonary vasodilator therapy as in this case; PEA, which constitutes major surgery to remove the chronic pulmonary emboli; and BPA, which is a minimally invasive procedure to improve pulmonary blood flow. Once identified and treated, the outcomes are often excellent [13,14].

In this instance, due to the combined presence of MEN1 complications and CTEPH, and their superimposed bearing on quality of life and disease burden, clinicians faced complex risk profiling with regard to invasive diagnostics and therapeutics. Another important consideration is the patient’s age and performance status; with MEN1 patients now living longer due to medical advancements [2] and PH now being associated with improved long-term outcomes due to developments in medical and surgical interventions [13], coupled with an ever-aging population in general, clinicians can expect to face further challenges when trying to optimise disease burden with invasive measures for these complex medical conditions.

In this clinical case, the patient discussed has approached the predicted average life expectancy age of MEN1, and there should be optimism that continued refinement in our understanding of the molecular pathways, genetic technology and emerging surveillance and therapeutic strategies will enable healthcare professionals to preserve quality of life during this time.

## Conclusions

MEN1 is a complex, multisystem genetic condition that requires lifelong close monitoring, screening and treatment planning; it is critical to involve a diverse group of clinicians spanning multiple specialities to optimise the patient journey, and be aware of local MEN1 specialist centres to ensure affected patients receive the necessary expertise in care. Its multisystem nature also contributes to high disease burden for most, and can lead to misdiagnosis or delayed identification of other significant healthcare issues. Therefore, clinicians should be vigilant and uphold a holistic approach to investigation of symptoms, whilst also considering the impact of chronic diseases on patients’ physical and mental health. There was a delay in CTEPH diagnosis in this case, which can have an excellent prognosis when identified early and treated by a specialist MDT; this stresses the importance of maintaining diagnostic attentiveness when new symptoms arise in patients with uncommon conditions. Furthermore, the increasing survival of MEN1 patients may result in more complex multimorbidity, and this highlights the need to manage rare and complex conditions with the appropriate expertise and MDT collaboration whilst having the overall aim of maintaining quality of life.

Of note, updated clinical guidelines have now revolutionised the MEN1 space and clinicians should be aware of the screening tools, diagnostic algorithms and treatment strategies available to them when suspecting or managing MEN1.
